# Potassium– and Lithium–Ammonia Intercalation into Excitonic Insulator Candidate Ta_2_NiSe_5_

**DOI:** 10.1021/acs.chemmater.4c02155

**Published:** 2024-09-20

**Authors:** Penny A. Hyde, Maxim Avdeev, Nicholas H. Rees, Simon J. Clarke

**Affiliations:** † Department of Chemistry, University of Oxford, Inorganic Chemistry Laboratory, South Parks Road, Oxford OX1 3QR, U.K.; ‡ Australian Centre for Neutron Scattering, Australian Nuclear Science and Technology Organisation, Lucas Heights, NSW 2234, Australia; § School of Chemistry, The University of Sydney, Sydney 2006, Australia

## Abstract

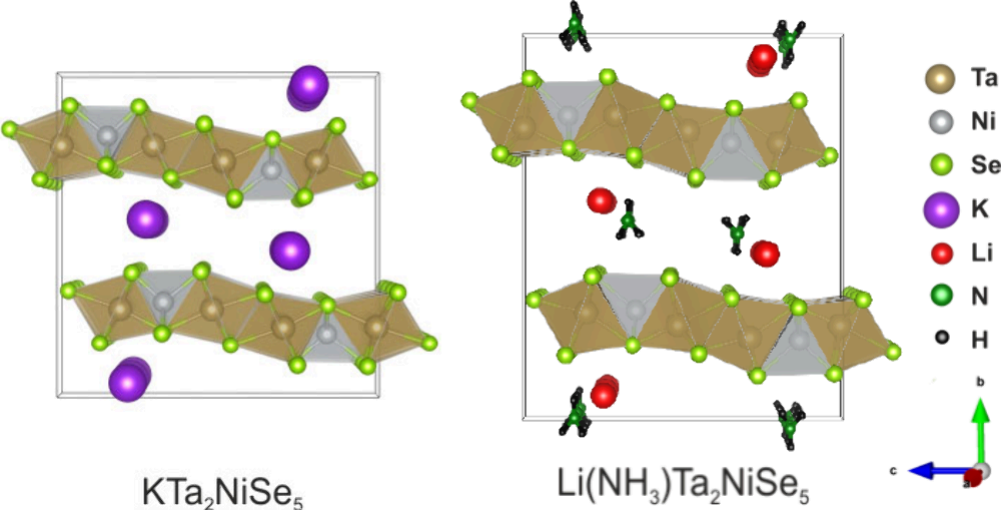

Two new reduced phases derived from the topical excitonic insulator candidate Ta_2_NiSe_5_ have been synthesized via the intercalation of lithium and potassium from solutions of the metals in liquid ammonia. Li(NH_3_)Ta_2_NiSe_5_ and KTa_2_NiSe_5_ both crystallize in orthorhombic space group *Pmnb* with the following lattice parameters: *a* = 3.5175(1) Å, *b* = 18.7828(7) Å, and *c* = 15.7520(3) Å and *a* = 3.50247(3) Å, *b* = 13.4053(4) Å, and *c* = 15.7396(2) Å, respectively. They have increased unit cell volumes of 48% and 31%, respectively, relative to that of Ta_2_NiSe_5_. Significant rearrangement of the transition metal selenide layers is observed in both intercalates compared to the parent phase. In Li(NH_3_)Ta_2_NiSe_5_, neutron diffraction experiments confirm the location of the light Li, N, and H atoms, and solid-state nuclear magnetic resonance (NMR) experiments show that H, N, and Li each occupy a single environment at ambient temperature on the NMR time scale. Magnetometry data show that both intercalates have increased magnetic susceptibilities relative to that of Ta_2_NiSe_5_, consistent with the injection of electrons during intercalation and an enhancement of the Pauli paramagnetism.

## Introduction

Intercalation is a versatile low temperature route to new materials unobtainable via other means and allows for the formation of kinetic products. A significant proportion of metal chalcogenides form layered structures, a consequence of the high polarizability of the chalcogenide ions,^1^ and are considered ideal candidates for intercalation chemistry due to their low dimensionality. The development of air-sensitive synthesis techniques in the 1980s resulted in a surge of synthesis of new layered ternary chalcogenides,^2−9^ in addition to improved characterization methods. Since then, transition metal chalcogenides have been studied extensively due to their unique physical properties and diverse structural chemistry. In addition to extensive intercalation chemistry of graphite,^10^ one of the first examples of intercalation chemistry was the insertion of Li into layered TiS_2_ to form Li_*x*_TiS_2_, which was used as a prototypical secondary battery system.^11^ As in that case, the intercalation of the reducing alkali metal with the insertion of electrons into the conduction band often transforms a semiconducting material into a metal. More recent examples include the topochemical insertion of Na or K into the layered thermoelectric compound Ta_2_PdS_6_,^12^ and the intercalation of Li and NH_3_ into the superconducting layered chalcogenide FeSe, which in this case changes the band structure of a metallic host material, as well as injecting electrons, and makes the superconducting ground state stable at a much higher temperature: enhancing the superconducting *T*_c_ by a factor of 5 to over 40 K.^13^

The narrow-band gap semiconductor, Ta_2_NiSe_5_ was discovered in the course of fundamental synthetic investigations by Ibers and co-workers and the preliminary physical property measurements were made in collaboration with DiSalvo and co-workers.^14^ The compound is of contemporary interest as a candidate for being a low-dimensional excitonic insulator.^15−17^ In the proposed excitonic insulator state, if the exciton binding energy is less than the band gap, excitons are formed spontaneously. Because the electron and hole pair (exciton) are strongly bound, the state is insulating. The transition to the excitonic insulating state can be understood as a Bose–Einstein Condensation (BEC) with the state arising from the formation of strongly bound charge-neutral pairs. It has been proposed as a high-carrier-mobility component of modern detectors for imaging applications,^18−21^ and it has recently been proposed that in the excited state it shows photonic time-crystalline behavior enabling THz amplification.^22^ It is a good example where the discovery of new compounds through exploratory chemical synthesis may lead to future applications as envisaged by DiSalvo.^23^

Ta_2_NiSe_5_ is a layered material, comprised of octahedral TaSe_6_ and tetrahedral NiSe_4_ polyhedra joined via edge sharing.^8^ The Ta_2_NiSe_5_ layers are stacked along the *b* axis, and are gently corrugated along the *c* direction. At room temperature Ta_2_NiSe_5_ behaves like a semiconductor, but above 550 K metallic behavior is observed.^14^ At 328 K, a structural phase transition, from a structure in space group *Cmcm* above this temperature to one in *C*2/*c* below, is observed. Tight-binding band structure calculations of the *Cmcm* structure indicate Ta_2_NiSe_5_ has a direct gap at the Γ point of the Brillouin zone.^24,25^ The symmetry-lowering distortion to *C*2/*c* accompanying the formation of the excitonic insulator phase allows mixing of the conduction and valence bands at Γ, which is proposed to account for the flattening of bands in this region.^26^

The possible oxidation state formalisms are Ta(IV)/Ni(II) or Ta(V)/Ni(0), with both configurations seeming plausible. The Ta(IV)/Ni(II) formalism may seem more chemically sensible, however the observed diamagnetism and the lack of evidence for a charge-density wave (CDW) distortion associated with the Ta(IV) 5*d*^1^ configuration suggests Ta(V)/Ni(0) is more appropriate. Structurally, the Ta–Ta separations are long (consistent with Ta(V)), while the Ta–Ni separation is short, consistent with a Ni(0) oxidation state stabilized by the neighboring Ta(V) acting as an electron acceptor.^24^ Electronically, there is no mixing of the states at the top of the Ni/Se-based valence band and the Ta-based conduction band because the states belong to different irreducible representations at Γ, and the appropriate formulation is Ta(V) and Ni(0). However, the situation seems finely balanced and the interpretation of ARPES data for the *C*2/*c* phase is suggestive of a Ta(IV) *d*^1^ state and an oxidized Ni3*d*^9^/Se-ligand hole state.^26−29^

Numerous reports have shown that the structure of Ta_2_NiSe_5_ is highly sensitive to temperature and applied pressure.^14,29,30^ The ambient-temperature and pressure phase crystallizes in *C*2*/c* and transforms to the *Cmcm* phase under applied hydrostatic pressure of approximately 2 GPa at ambient temperature, which is the same as the high-temperature/ambient-pressure phase. It then transforms to a *Pmnm* phase at about 3 GPa at ambient temperature by a relative sliding of the layers, resulting in a loss of lattice centring to produce an AA-type stacking instead of an AB-type stacking. This orthorhombic *Pmnm* phase also undergoes a transition on cooling at high pressure to a monoclinic phase in *P*2*/n* which Nakano et al.^30^ suggest may also be an excitonic insulator phase.

Intercalation of lithium into Ta_2_NiSe_5_ has previously been reported^31,32^ using *n*-butyllithium to form LiTa_2_NiSe_5_ motivated by the injection of electrons into the conduction band to drive the system into the metallic regime. Significant rearrangement of the stacking of the transition metal selenide slabs occurs upon intercalation, and no structural transition, akin to the orthorhombic to monoclinic distortion accompanied by a transition to insulating behavior reported for Ta_2_NiSe_5_, was observed down to 100 K.^32^ Here we present the reduced phases KTa_2_NiSe_5_ and Li(NH_3_)Ta_2_NiSe_5_ synthesized using intercalation and characterized using high-resolution PXRD, neutron powder diffraction, solid state NMR spectroscopy and magnetometry. We compare and contrast these new reduced phases with the host Ta_2_NiSe_5_ and its lithium intercalate LiTa_2_NiSe_5_.^8,32^

## Experimental Section

### Synthesis

All syntheses were carried out in a Glovebox Technology argon-filled dry glovebox with an O_2_ content below 1 ppm or on a Schlenk line. Polycrystalline samples of Ta_2_NiSe_5_ were synthesized by grinding together tantalum powder (Alfa Aesar 99.97%), nickel powder (Alfa Aesar 99.9%) and selenium powder (Alfa Aesar 99.999%) in stoichiometric amounts using an agate pestle and mortar until homogeneous. The mixture was then sealed inside an evacuated silica tube and heated at 750 °C for 7 days (ramping rate of 5 °C min^–1^) before being allowed to cool at the natural rate of the furnace. The resulting powder was reground and pressed into a pellet before reheating to 700 °C for 48 h and was then left to cool at the natural rate of the furnace. This second step was often required to improve the crystallinity of the samples.

Lithium-ammonia and potassium intercalates were synthesized by adding Ta_2_NiSe_5_ powder to a Schlenk tube under argon, along with a magnetic stirrer bar and a stoichiometric amount of Li or K metal (Sigma-Aldrich; 99%) to give the target phase *A*Ta_2_NiSe_5_, (*A* = Li, K). Approximately 10 cm^3^ of ammonia was condensed onto the reagents using a Schlenk line connected to a cylinder of ammonia (BOC; 99.98%) and with the Schlenk tube cooled to approximately −78 °C in a bath of isopropanol and dry ice. The suspension was left to stir until all the ammonia had evaporated, then the remaining solid was dried under dynamic vacuum for 20 min. (Caution: ammonia is volatile and toxic. The synthesis was performed in a fume hood and at all times the liquid-ammonia-containing vessel was open to a mercury bubbler to avoid pressures exceeding 1 bar in the reaction vessel). The products were highly air and moisture sensitive and were sequestered from air during measurements and handling.

### Diffraction Measurements

Laboratory PXRD data was collected using a Bruker D8 Advance Eco diffractometer (Cu K_α_ radiation) in order to follow the reaction between heating and intercalation steps. For detailed structural refinement, data was collected on beamline I11^33^ at the Diamond Light Source using 1.5 min scans with the MYTHEN Position Sensitive Detector (PSD), using Si-calibrated 0.82 Å X-rays. Powder Neutron Diffraction (PND) measurements at ambient temperature were made on the Li/NH_3_ intercalate using the Echidna instrument at the Australian Centre for Neutron Scattering (ACNS), Australian Nuclear Science and Technology Organization (ANSTO), Sydney, Australia.^34^ A sample of approximately 1.5 g in mass was loaded in an Ar glovebox into a 6 mm diameter vanadium can and sealed using indium gaskets. Refinement of the structural models against the diffraction data was carried out using the TOPAS Academic V6 software.^35^ Anisotropic peak broadening was accounted for by the method of Stephens^36^ as implemented in TOPAS.

### Magnetometry

Magnetic susceptibility measurements were made using a Quantum Design MPMS-3 SQUID magnetometer. Accurately weighed samples of around 30 mg in mass were loaded into gelatin capsules, which were secured inside plastic straws which were loaded into the instrument.

### Chemical Analysis

Inductively coupled-plasma mass spectrometry (ICP-MS) was used to determine the Li content of Li-containing samples. Approximately 5 mg of the sample was dissolved in 20 mL of HNO_3_ (conc.): HCl (12 M) in a 19:1 volume ratio and diluted with deionized water to make a 2 vol % solution of the original concentrated acids. A PerkinElmer NexION 350D ICP-MS instrument at the Department of Geography, University of Oxford, was used to determine the concentrations of Li and Ni. CHN analysis using the Dumas combustion method was performed by Elemental Microanalysis Ltd., Okehampton, UK.

### Solid State NMR Spectroscopy

All nuclear magnetic resonance (NMR) measurements were made using a Bruker Avance III-HD spectrometer equipped with a 9.4 T wide-bore magnet and a 4 mm magic angle spinning (MAS) probe. ^6^Li spectra were measured at 243, 273, 293, and 313 K with a spinning rate of 8 kHz. For the ^6^Li measurement 2000 scans were acquired with a pulse delay of 18 s. Spectra were externally referenced to LiCl in H_2_O (0 ppm). The ambient temperature ^1^H spectrum was acquired with 16 scans, using a background suppression sequence and a pulse delay of 4 s. The spectra were externally referenced to adamantane (chemical shift, δ, of 1.82 ppm relative to Tetramethylsilane (TMS) at 0 ppm). Natural abundance ^15^N Cross-Polarization Magic-Angle Spinning (CPMAS) measurements were made at ambient temperature with a spinning rate of 10 kHz, a contact time of 4.5 ms, pulse delay of 1.6 and 45000 scans. Spectra were externally referenced to glycine (δ 33.4 ppm relative to ^15^NH_4_NO_3_ at 0 ppm).

## Results and Discussion

### Structural Refinement

**Figure 1:**
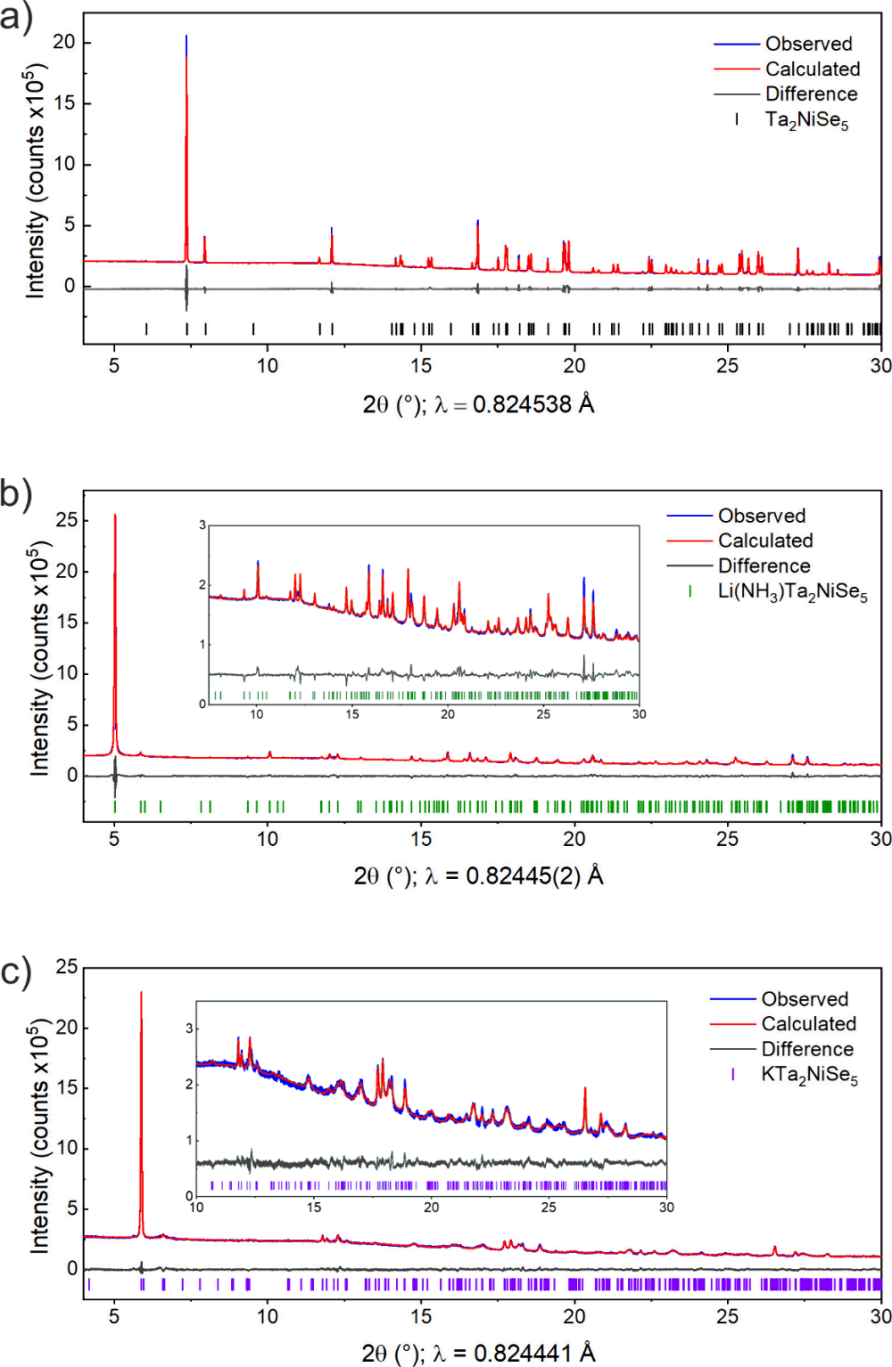
Rietveld refinements of (a) Ta_2_NiSe_5_, (b) Li(NH_3_)Ta_2_NiSe_5_, and (c) KTa_2_NiSe_5_ measured at 300 K on the PSD detector at I11 showing the observed (blue), calculated (red), and difference (gray) curves. *R*_wp_ values of 2.23%, 2.11%, and 2.74%, respectively.

The PXRD patterns of the intercalated phases have some visual similarities to that of the parent compound Ta_2_NiSe_5_. An increased intensity of the low-angle 020 reflection was evident in the intercalates, as shown in Figure 1, a relatively common observation in layered materials. This was accounted for by employing a spherical harmonic type preferred orientation correction. The patterns of the potassium and lithium-ammonia intercalates could be well indexed to orthorhombic unit cells, *a* = 3.5928(1) Å, *b* = 16.0497(1) Å, *c* = 15.8774(4) Å and *a* = 3.5175(1) Å, *b* = 18.7828(7) Å, *c* = 15.7520(3) Å respectively. Both unit cells have similar *a* and *c* parameters to the parent Ta_2_NiSe_5_ phase, but have significantly elongated *b* parameters along the stacking direction relative to that of the parent, which gives increased unit cell volumes by 31% and 48% for KTa_2_NiSe_5_ and Li(NH_3_)Ta_2_NiSe_5_ respectively compared with Ta_2_NiSe_5_. A rigid body approach, as described in the structure solution of LiTa_2_NiSe_5_,^32^ was adopted to take advantage of the layered nature of the structure and made the assumption that the layers would remain intact during the low temperature intercalation experiments, but might move substantially relative to one another.^32^ A model was constructed in *P*1 containing the layers similar to those in Ta_2_NiSe_5_. These were stacked within the new expanded cells identified above. Atomic positions were constrained so there was initially no refinement of relative atomic positions within each layer. One layer was kept fixed and the position of the other was refined in the *x* and *z* directions relative to the first. The displacement in the *y* direction (i.e., the stacking direction) was fixed to zero so that the layers were equally spaced. The refined relative displacements of the two layers in the unit cell compared with the case of the parent Ta_2_NiSe_5_ were (0.5, 0, – 0.1703(7)) for the potassium intercalate, and (0.5, 0, – 0.1885(3)) for the lithium-ammonia intercalate. As reported for LiTa_2_NiSe_5_, the displacements in *x* (i.e., along the short axis) from this rigid-body refinement are found to be 0.5–i.e. alternating layers are no longer offset along the *a* direction and are not related by the *C*-centring translation. The layers become displaced slightly relative to one another along the *c* direction. The resulting structures obtained from the rigid-body refinements in *P*1 can then be described in *Pmnb* (a setting of space group No. 62). The final models were produced by lifting the rigid body constraints and allowing atomic positions to freely refine in the *Pmnb* cell. To produce a complete model of KTa_2_NiSe_5_, potassium was added at approximately (0.25, 0.5, 0.3), based on the identification of a positive scattering center in the TOPAS-generated Fourier map, and this was allowed to freely refine in the final model. Due to the large number of parameters required to describe the structures and the relative low intensities of the PXRD patterns in the region 2θ > 7°, atomic displacement parameters of all Se sites were constrained to be equal in the refinement of both intercalates. The position of Ni was fixed in the model of KTa_2_NiSe_5_ for the same reasons. Refined parameters of KTa_2_NiSe_5_ and Li(NH_3_)Ta_2_NiSe_5_ are given in Tables 1 and 2 respectively. In the diffraction patterns, 0*k*0 reflections (*b* is the stacking direction) are sharp, while reflections with large *h* and/or *l* values are somewhat broader, and this was accounted for using the Stephens approach.^36^

**Table 1:** Refined Parameters from the Rietveld Fit of the PXRD Pattern of KTa_2_NiSe_5_ Collected Using the PSD at I11^a^

KTa_2_NiSe_5_, RMM = 854.49 g mol^–1^, *Z* = 4	
diffractometer	I11 (PSD)
wavelength (Å)	0.824441
temperature (K)	300
space group	*Pmnb* (62)
*a* (Å)	3.5928(1)
*b* (Å)	16.0497(1)
*c* (Å)	15.8774(8)
*V* (Å^3^)	915.55(7)

atom	site	*x*	*y*	*z*	occupancy	*U*_iso_ (Å^2^)
Ta1	4*c*	0.25	0.2207(4)	0.0193(4)	1	0.023(3)•
Ta2	4*c*	0.25	0.2027(4)	0.3204(5)	1	0.023(3)•
Ni	4*c*	0.25	0.3099*	0.6750*	1	0.023(3)•
Se1	4*c*	0.25	0.8971(9)	0.952(1)	1	0.032(4)†
Se2	4*c*	0.25	0.920(1)	0.7101(9)	1	0.032(4)†
Se3	4*c*	0.25	0.1790(8)	0.8628(9)	1	0.032(4)†
Se4	4*c*	0.25	0.1620(9)	0.477(1)	1	0.032(4)†
Se5	4*c*	0.25	0.296(1)	0.170(2)	1	0.032(4)†
K	4*c*	0.25	0.488(2)	0.331(8)	1.01(7)	0.082(2)

a Values marked with an asterisk were fixed. Values marked with a bullet or dagger were constrained to refine to the same value.

**Table 2:** Refined Parameters from the Rietveld Fit of the PXRD and PND (in parentheses) Patterns of Li(NH_3_)Ta_2_NiSe_5_^a^

Li(NH_3_)Ta_2_NiSe_5_, RMM = 838.35 g mol^–1^, *Z* = 4	
diffractometer	I11 (PSD) (Diamond) (Echidna(ACNS))
wavelength (Å)	0.82445(2) (1.622)
temperature (K)	300 (300)
space group	*Pmnb* (62)
*a* (Å)	3.51753(5) (3.5146(3))
*b* (Å)	18.7828(7) (18.799(4))
*c* (Å)	15.7520(3) (15.712(1))
*V* (Å^3^)	1040.72(5) (1038.2(3))

atom	site	*x*	*y*	*z*	occupancy	*U*_iso_ (Å^2^)
Ta1	4*c*	0.25 (0.25)	0.2297(4) (0.235(2))	0.0158(3) (0.024(1))	1	0.0097(18) (0.026(5))
Ta2	4*c*	0.25 (0.25)	0.2219(3) (0.224(2))	0.2957(2) (0.293(1))	1	0.0046(16) (0.019(4))
Ni	4*c*	0.25 (0.25)	0.2795(6) (0.289(1))	0.6604(9) (0.658(1))	1	0.0052(31) (0.044(4))
Se1	4*c*	0.25 (0.25)	0.8550(6) (0.868(1))	0.9593(7) (0.9606(7))	1	0.0041(13)** (0.004(1))†
Se2	4*c*	0.25 (0.25)	0.8682(7) (0.874(1))	0.7319(6) (0.7293(7))	1	0.0041(13)** (0.004(1))†
Se3	4*c*	0.25 (0.25)	0.1803(5) (0.1759(1))	0.8555(7) (0.8514(8))	1	0.0041(13)** (0.004(1))†
Se4	4*c*	0.25 (0.25)	0.1871(6) (0.180(1))	0.4544(6) (0.4496(7))	1	0.0041(13)** (0.004(1))†
Se5	4*c*	0.25 (0.25)	0.3018(5) (0.291(1))	0.1578(7) (0.1496(8))	1	0.0041(13)** (0.004(1))†
Li	(4*c*)	(0.75)	(0.432(1))	(0.245(3))	1.02(4)	(0.036(6))
N	(4*c*)	(0.25)	(0.483(3))	(0.330(2))	1*	(0.046(9))
H1	(8*d*)	(0.640(10))	(0.549(4))	(0.641(2))	0.56(6)	(0.0395(4))‡
H2	(8*d*)	(0.641(9))	(0.473(5))	(0.689(5))	0.53(7)	(0.0395(4))‡
H3	(8*d*)	(0.649(9))	(0.462(5))	(0.622(7))	0.55(6)	(0.0395(4))‡

a Values marked with an asterisk were fixed. Atomic displacement parameters marked with two asterisks, a dagger, or a double dagger were constrained to refine to the same value.

PND experiments were performed in order to locate the light atoms; Li, N and H. The Rietveld refinement against room temperature data from Echidna (ACNS) was consistent with the *Pmnb* model of the transition metal selenide layers generated from the refinement against PXRD data. An interlayer scattering center was identified at (0.25, 0.483(3), 0.330(2)) using TOPAS-generated Fourier maps. From visual inspection, this site had a co-ordination environment of six surrounding Se atoms at a distance of approximately 3.7 Å. Given that lithium-ammonia intercalates of FeSe^13^ and Bi_2_Se_3_^37^ report H–Se and N–H distances of approximately 2.7 and 1 Å respectively, this site satisfied the co-ordination requirements of NH_*x*_ (*x* = 2, 3). N was assigned to this position, H sites were then added at three different crystallographic positions surrounding N consistent with these H–Se and N–H distances and allowed to refine. Due to the symmetry of the structure six hydrogen atoms are generated surrounding each N. The occupancies of these sites refined to be approximately 50% occupied, consistent with the stoichiometry of ammonia, and with orientational disorder of the NH_3_ molecule. Initially the H positions were allowed to freely refine, however the addition of a soft restraint to give an approximate N–H bond length of 1 Å was found to aid stability in the initial stages of the refinement. Li was then added to several sites at approximately 2.4 Å from the refined N position, but only refined to a chemically sensible position at (0.25, 0.568(1), 0.755(3)), which is translated by *a*/2 relative to the nearest N atom. In the final stages of the refinement Li, N and H positions were refined independently of one another. This resulted in a Li–N distance of 2.41(7) Å and an average N–H bond length of 1.04(8) Å, The average H occupancy refined to 0.55(4) (Table 2), which gives a Li:N:H ratio of 1:1:3.3(1). The H–Se hydrogen bonds are found to range from 2.61(3) – 2.73(2) Å and the average N–H–Se angle is 172(2)°. All bond distances and angles are consistent with those in the lithium-ammonia intercalates of FeSe^13^ and Bi_2_Se_3_,^37^ the latter of which reports weak H–Se bonds of approximately 2.8 Å and a near linear N–H–Se angle. The two Li–N distances of 2.41(7) Å, are within error of the Li–N bond length in crystalline LiNH_2_ of 2.35 Å^38^ suggesting there is significant bonding between the two species Li^+^ and NH_2/3_. The shortest Li–Se distance is 2.64(5) Å and two further Li–Se distances of 3.51(7) Å complete the distorted Li coordination sphere. CHN combustion analysis gave a N:H ratio as 2.62(7). Chemical analysis (ICP-MS) gives a Li:Ni ratio of 1.10(4):1, which is in good agreement with the freely refined Li occupancy of 1.02(4) which we interpret as a fully occupied Li site. Combination of the combustion analysis and ICP-MS results gives a stoichiometry of Li_1.10(4)_(NH_2.62(7)_)Ta_2_NiSe_5_, while the PND refinement gives a stoichiometry of Li_1.02(4)_(NH_3.3(1)_)Ta_2_NiSe_5_. Given the experimental uncertainties in these techniques, the results are consistent with the stoichiometry Li(NH_3_)Ta_2_NiSe_5_ and reduction of the parent Ta_2_NiSe_5_ phase, although from the chemical analysis result we cannot rule out partial reduction of NH_3_ to NH_2_^–^.

**Figure 2:**
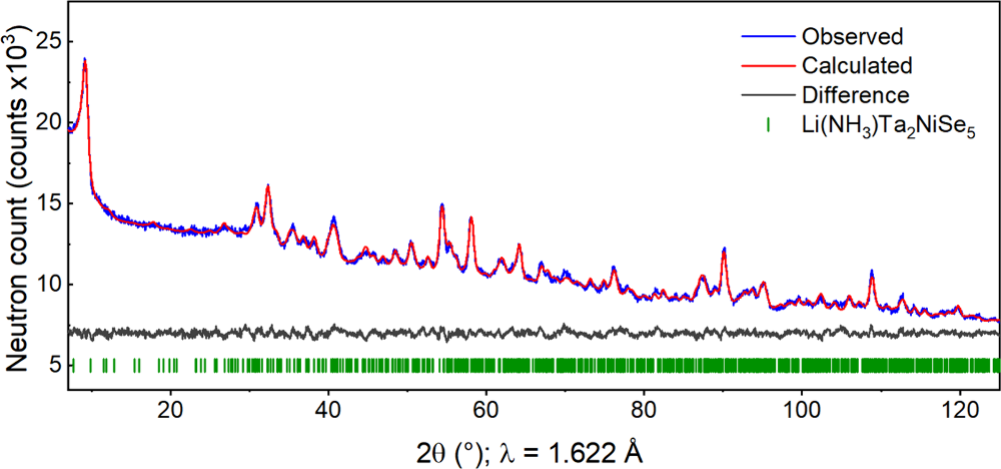
Rietveld refinement against PND data of Li(NH_3_)Ta_2_NiSe_5_ collected on the Echidna instrument at ACNS, showing the observed (blue), calculated (red), and difference (gray) curves. *R*_wp_ = 1.43%.

**Figure 3:**
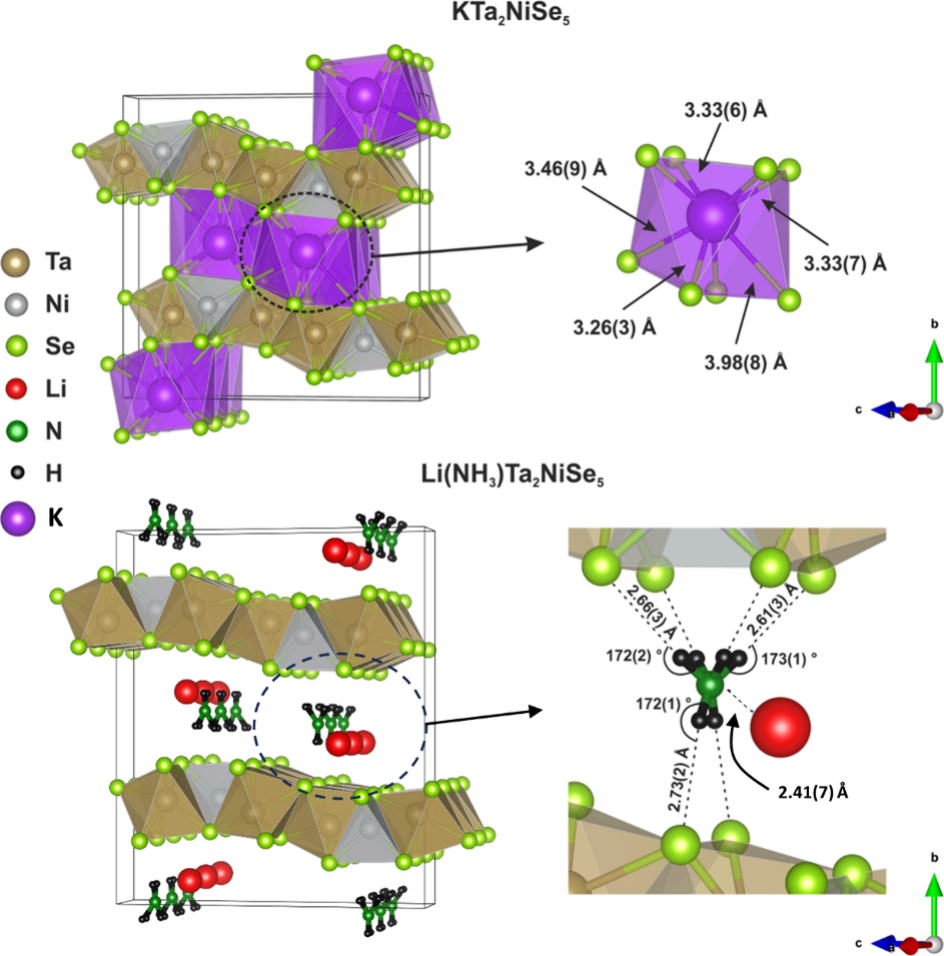
Structural models of KTa_2_NiSe_5_ (top) and Li(NH_3_)Ta_2_NiSe_5_ (bottom) from the refinement against PXRD and PND data.

The Rietveld fit of the PND data is shown in Figure 2, and the final structural models of both intercalates are shown in Figure 3.

The general structure of the Ta–Ni–Se layers is maintained in both intercalates. In KTa_2_NiSe_5_, potassium is found to occupy 8-coordinate bicapped trigonal prism sites with an average K–Se bond length of 3.41(3) Å. This is consistent with K–Se bond length values reported in the literature, which range from 3.33 Å for the tetrahedral environment in K_2_Se^39^ to 3.42–3.53 Å for the 8-coordinate K sites in KFeSe_2_.^40^ There is one long K–Se bond which has been included in the formal coordination sphere with a bond distance of 3.98(8) Å, as reports of long K–Se bond lengths exist in the literature, especially for more unusual coordination geometries. For example, bonds of up to 3.95 Å have been reported for K_3_(FeSe_2_)_2_.^41^

Table 3 compares the lattice parameters and weighted average bond lengths of Ta_2_NiSe_5_ and the two intercalated phases, Li(NH_3_)Ta_2_NiSe_5_ and KTa_2_NiSe_5_ along with LiTa_2_NiSe_5_.^32^ The refined structural models show elongation of the Ta–Se and Ni–Se bonds in the intercalates relative to Ta_2_NiSe_5_, indicating some partial reduction of both transition metals occurs upon intercalation which is consistent with the injection of electrons. The larger change in the potassium intercalate compared with the lithium-ammonia intercalate, could be consistent with partial reduction of ammonia to amide (and hence less reduction of Ta) as suggested by the chemical analysis, however the change, compared with Ta_2_NiSe_5_, in the case of LiTa_2_NiSe_5_^32^ is similar to that in Li(NH_3_)Ta_2_NiSe_5_, suggesting that the larger change in the case of the KTa_2_NiSe_5_ may be more likely governed mainly by the steric demands of the larger K^+^ ion than the electron count.

**Table 3:** Refined Lattice Parameters and Weighted Average Bond Lengths of Ta_2_NiSe_5_, KTa_2_NiSe_5_, and Li(NH_3_)Ta_2_NiSe_5_ from the Rietveld Refinement against PXRD and PND Data

	Ta_2_NiSe_5_	LiTa_2_NiSe_5_^32^	KTa_2_NiSe_5_	Li(NH_3_)Ta_2_NiSe_5_	
	X-ray radiation	X-ray radiation	X-ray radiation	X-ray radiation	neutron radiation
*a* (Å)	3.4945(1)	3.50247(3)	3.5928(1)	3.5175(1)	3.5167(3)
*b* (Å)	12.8294(1)	13.4053(4)	16.0497(1)	18.7828(7)	18.831(4)
*c* (Å)	15.6431(3)	15.7396(2)	15.8774(8)	15.7520(3)	15.713(1)
β (deg)	90.53(1)	90	90	90	90
*V* (Å^3^)	701.29(1)	739.00(3)	915.55(7)	1040.72(5)	1040.6(3)
space group	*C*2/*c* (15)	*Pmnb* (62)	*Pmnb* (62)	*Pmnb* (62)	*Pmnb* (62)
(Ta1–Se)_average_ (Å)	2.575	2.600	2.652	2.582	2.638
(Ta2–Se)_average_ (Å)	2.607	2.593	2.720	2.588	2.590
(Ni–Se)_average_ (Å)	2.360	2.369	2.485	2.35	2.42
ΔIS (Å)^a^	0	0.576	1.6102	2.9767	2.985

a ΔIS is the change in interlayer spacing (change in *b*/2) relative to Ta_2_NiSe_5_.

### NMR Spectroscopy

**Figure 4:**
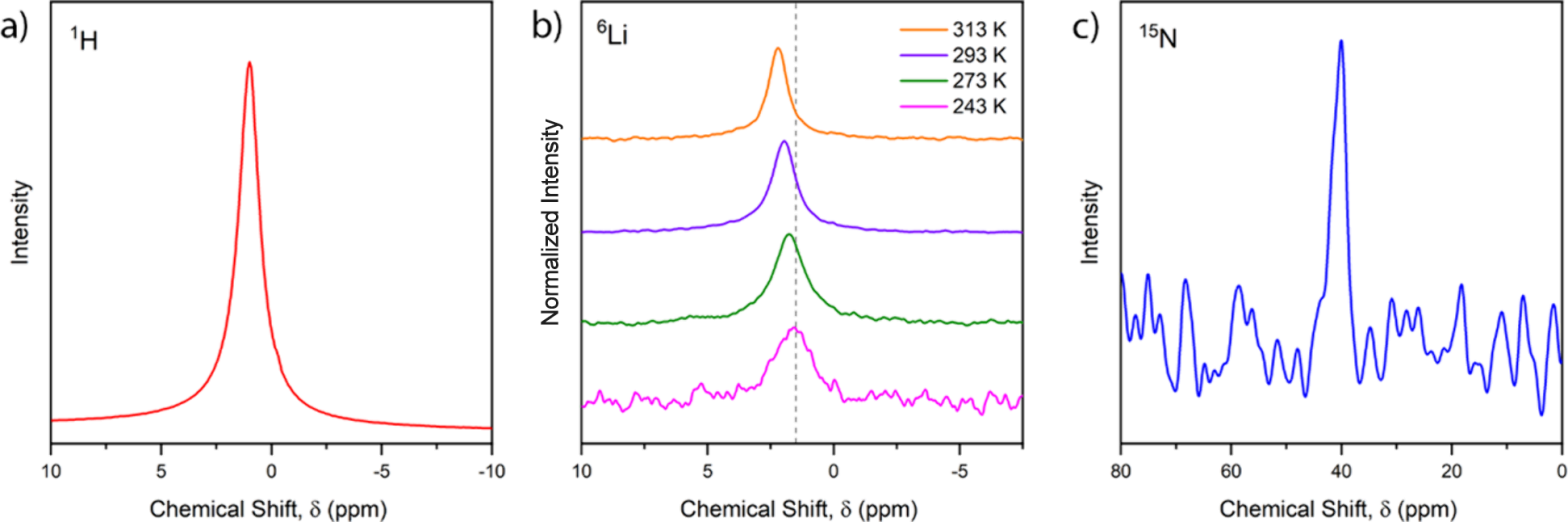
(a) Ambient temperature magic-angle spinning (MAS) ^1^H NMR spectrum, (b) variable temperature static ^6^Li NMR spectra, and (c) ambient temperature static ^15^N NMR spectrum of Li(NH_3_)Ta_2_NiSe_5_.

Solid-State NMR experiments were performed to probe the local environments of the light atoms, H, Li and N, in Li(NH_3_)Ta_2_NiSe_5_. Spectra are shown in Figure 4. The ambient temperature magic-angle spinning (MAS) ^1^H and ^15^N NMR spectra both show single proton and N environments, on the NMR time scale. This agrees with our analysis of PND data, which reveals a single NH_*x*_ site. While the diffraction data are interpreted using three distinct H sites, rapid exchange between these sites as a consequence of rapid tumbling of the ammonia/amide molecules will result in a single averaged resonance in the NMR spectrum.

Variable temperature MAS ^6^Li NMR measurements indicate a single Li environment at 298 K on the NMR time scale, consistent with the single site deduced from our structural model obtained from fitting the PND pattern. Low temperature measurements show a subtle peak shift to lower chemical shift δ. We hypothesize this is a temperature-dependent Knight shift, observed in metallic compounds arising from the Pauli paramagnetism of conduction electrons, and proportional to the electronic density of states at the Fermi level.^42,43^ The ^6^Li resonance broadens as the temperature is reduced, which could indicate a fluctional process which would require lower temperature measurements for further investigation.

### SQUID Magnetometry

Ambient temperature magnetization isotherms measured using SQUID magnetometry reveal that the parent and intercalate compounds are bulk diamagnets. At low fields, all isotherms reveal small positive magnetizations from 0–1 T. This is indicative of a small ferromagnetic impurity and has been attributed to the presence of elemental Ni in trace amounts well below the detection limit of X-ray powder diffraction. The susceptibilities were determined by measuring the gradient as a function of temperature in the linear regions of the magnetization isotherms at higher fields. This was performed by subtracting the magnetization measured at 4 T from that measured at 3 T as a function of temperature.

**Figure 5:**
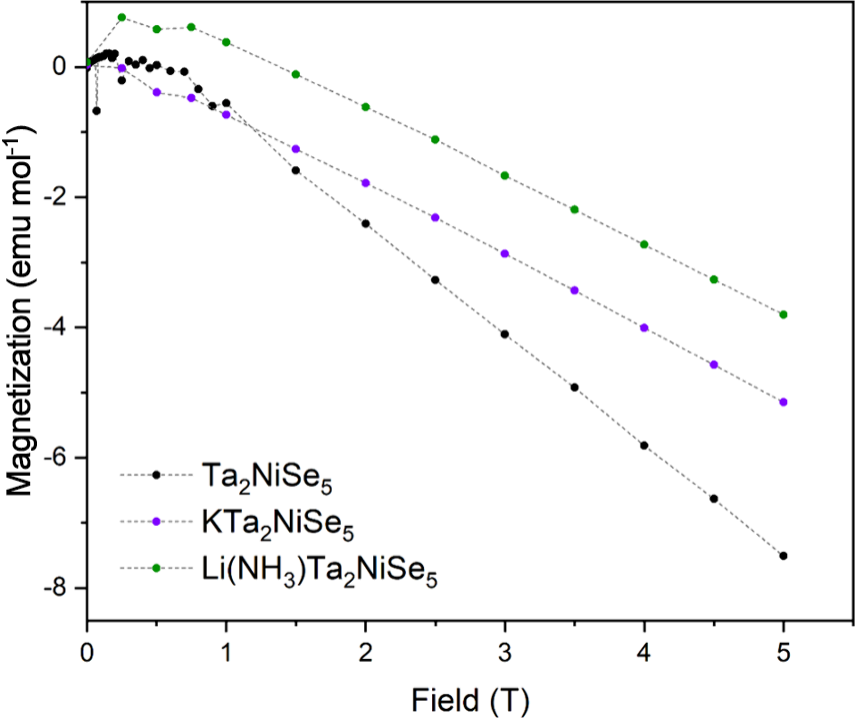
Magnetization isotherms of Ta_2_NiSe_5_, KTa_2_NiSe_5_, and Li(NH_3_)Ta_2_NiSe_5_ measured at 300 K over the range of 0–5 T.

Figure 5 reveals that a 34–37% reduction in the diamagnetic susceptibility occurs, from −1.695(7) × 10^–4^ emu mol^–1^ in Ta_2_NiSe_5_ (DiSalvo et al. report a susceptibility of −1.028 × 10^–4^ emu mol^–1^ for Ta_2_NiSe_5_ at 300 K^14^) to −1.126(6) × 10^–4^ emu mol^–1^ in KTa_2_NiSe_5_ and −1.071(2) × 10^–4^ emu mol^–1^ in Li(NH_3_)Ta_2_NiSe_5_, upon intercalation. The experimental values are significantly less negative than the core diamagnetic contributions calculated from standard tables^44^ (−2.8 × 10^–4^ emu mol^–1^, – 3.0 × 10^–4^ emu mol^–1^ for Ta_2_NiSe_5_ and both intercalates respectively). Thus the parent Ta_2_NiSe_5_ has some temperature-independent paramagnetic contribution and this suggests that the intercalates are both Pauli paramagnets with a temperature-independent susceptibility enhanced by the injection of electrons to increase the density of states at the Fermi Level. There was no temperature dependence of the intrinsic susceptibility, (see Figures S1 and S2 for KTa_2_NiSe_5_ and Li(NH_3_)Ta_2_NiSe_5_ respectively). This is consistent with the behavior of the previously reported Li intercalate, LiTa_2_NiSe_5_.^32^Table 4 shows the experimentally measured magnetic susceptibilities and paramagnetic contribution to the magnetic susceptibilities (calculated by subtracting the core diamagnetism from standard tables from the experimentally measured susceptibilities) of Ta_2_NiSe_5_, previously reported intercalate LiTa_2_NiSe_5_^32^ and both intercalates presented here. The corrected susceptibilities suggest the Li, K and Li(NH_3_) intercalates undergo comparable degrees of reduction, although the precise values will depend on the details of the band structure, which will correlate with local coordination environments, as well as the electron count. Additional variable temperature measurements were performed in a field of 50 Oe and revealed no observable superconducting transition down to 2 K, the lower limit of our instrument.

**Table 4:** Experimentally Determined Magnetic Susceptibilities of Ta_2_NiSe_5_ and Its Intercalates at Room Temperature

compound	experimental magnetic susceptibility (emu mol^–1^)	corrected magnetic susceptibility (emu mol^–1^)^a^
Ta_2_NiSe_5_^32^	–1.695(7) × 10^–4^	1.105(7) × 10^–4^
LiTa_2_NiSe_5_^32^	–1.254(4) × 10^–4^	1.556(4) × 10^–4^
KTa_2_NiSe_5_	–1.126(6) × 10^–4^	1.884(6) × 10^–4^
Li(NH_3_)Ta_2_NiSe_5_	–1.071(2) × 10^–4^	1.929(2) × 10^–4^

a The corrected magnetic susceptibility removes the diamagnetic contribution to the measured susceptibility and was calculated by subtracting the core diamagnetism from standard tables^44^ from the experimentally measured magnetic susceptibility.

## Conclusions

We have synthesized and characterized the phases Li(NH_3_)Ta_2_NiSe_5_ and KTa_2_NiSe_5_, obtained by the intercalation into the topical excitonic insulator candidate Ta_2_NiSe_5_ using alkali metals (*A* = Li, K) dissolved in ammonia. The transition metal selenide layers remain intact during the intercalation process, but undergo significant rearrangement with respect to one another, resulting in the loss of *C*-centring. Large unit cell expansions of 48% and 31% for *A* = Li, K respectively are observed. The larger than expected expansion for *A* = Li indicates the cointercalation of NH_3_ had occurred, presumably because Li^+^ is so strongly solvated by NH_3_ which was confirmed through PND, ^15^N and ^1^H NMR spectroscopy and elemental analysis. Both potassium and ammonia occupy similar regions of the unit cell in the respective intercalates. SQUID magnetometry shows that both intercalates are diamagnetic, but show significant reduction in diamagnetic susceptibility due to increased competing Pauli paramagnetism through injection of electrons into the conduction band, increasing the density of states at the Fermi level. This work demonstrates the versatility of the intercalation route to produce a wide range of new materials with metal ion and sometimes molecular cointercalants and tune the properties of potential device materials such as Ta_2_NiSe_5_.

## Supporting Information Available

Additional tables of refinement parameters and a figure showing magnetometry data (PDF)
