# Supplementary material for: Potassium– and Lithium–Ammonia Intercalation into Excitonic Insulator Candidate Ta2NiSe5
Source: Chem Mater. 2024 Sep 20;36(19):9939–46. doi: 10.1021/acs.chemmater.4c02155 (PMC11467832; doi:10.1021/acs.chemmater.4c02155)
Supplement: Supplementary file 1 — cm4c02155_si_001.pdf [file cm4c02155_si_001.pdf]

Potassium and Lithium-ammonia Intercalation into Excitonic Insulator Candidate Ta_2NiSe_5

Penny A. Hyde,[a] Maxim Avdeev,[b,c] Nicholas H. Rees,[a] Simon J. Clarke*[a]

[a]*Department of Chemistry, University of Oxford, Inorganic Chemistry Laboratory, South Parks Road, Oxford OX1 3QR, UK*

[b]*Australian Centre for Neutron Scattering, Australian Nuclear Science and Technology Organisation, Lucas Heights, NSW 2234, Australia*

[c]*School of Chemistry, The University of Sydney, Sydney 2006, Australia*

Supporting Information

*Corresponding author: simon.clarke@chem.ox.ac.uk

Table S1. Experimental and refined parameters of $Li(NH_3)Ta_2NiSe_5$ from the PXRD pattern collected on the I11 beamline and the PND pattern collected on Echidna, and KTa_2NiSe_5 from the PXRD pattern collected on the I11 beamline.

	**$Li(NH_3)Ta_2NiSe_5$**		**KTa_2NiSe_5**
Diffractometer	Echidna (ACNS)	I11 (PSD)	I11 (PSD)
Radiation	Neutron	X-ray	X-ray
Wavelength (Å)	1.622	0.82445(2)	0.824441
d-space range (Å)	0.82-23.4	0.57-22.7	0.57-22.7
Temperature (K)		300	
Crystal system		Orthorhombic	
Space group		*Pmnb* (62)	
a (Å)	3.5146(3)	3.5175(1)	3.5928(1)
b (Å)	18.799(4)	18.7828(7)	16.0497(1)
c (Å)	15.712(1)	15.7520(3)	15.8774(8)
V ($Å^3$)	1038.2(3)	1040.72(5)	915.55(7)
RMM ($g\ mol^{-1}$)	838.35		854.49
Z	4	4	4
χ^2	1.15	134.07	125.04
R_p	1.11	2.03	2.16
R_{wp}	1.43	2.11	2.74

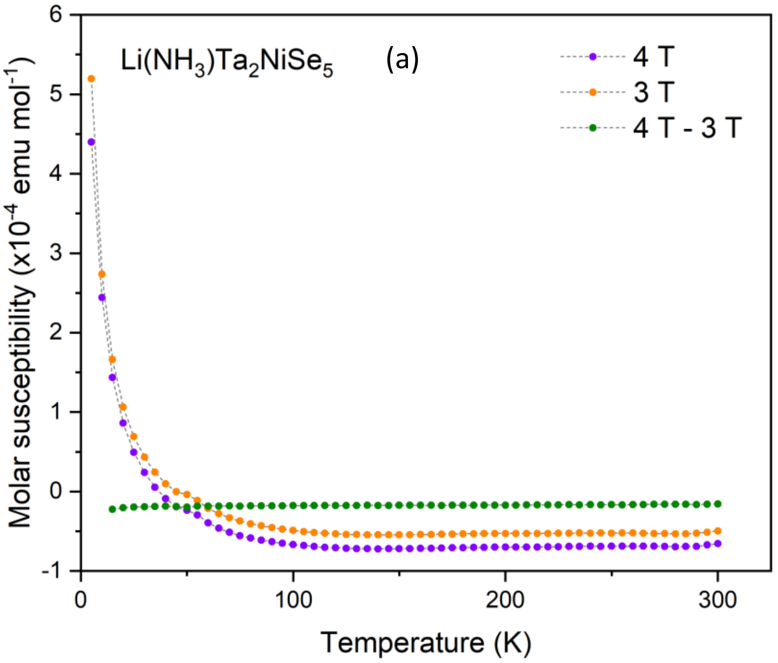

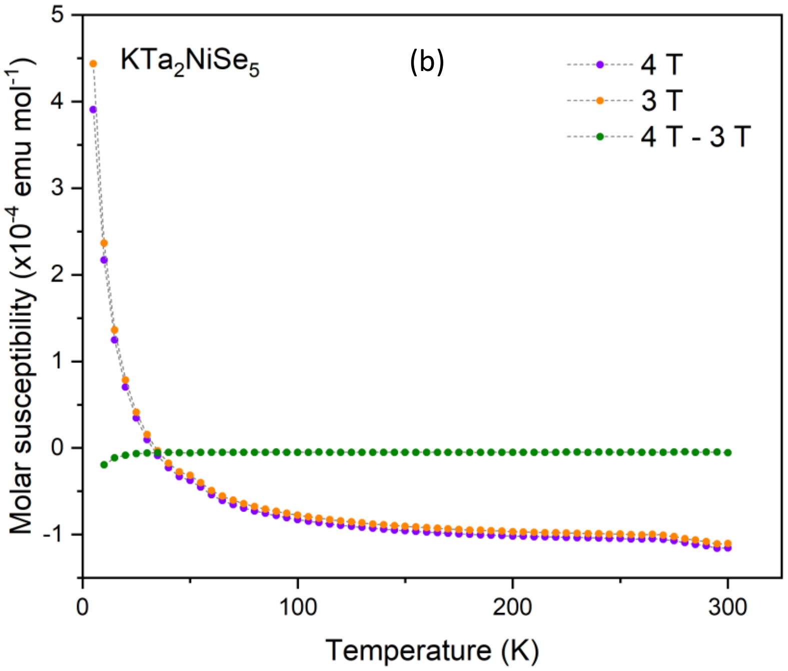

Figure S1. Susceptibility vs temperature curves of for **(a)** $Li(NH_3)Ta_2NiSe_5$ and **(b)** KTa_2NiSe_5. Data were collected applied fields of 4T (purple) and 3T (orange). The intrinsic susceptibility obtained by subtraction of the 3T data from the 4T data at each temperature is given in green, and this corrects for a minuscule magnetic impurity evident in the magnetisation isotherms in Figure 5 of the main article.

Note for Tables

In the Rietveld refinement the function S_y is minimised

$$S_y = \sum_i w_i (y_i - y_{ci})^2$$

where y_i is the observed, and y_{ci} the calculated intensity at point i and w_i is the weighting factor, defined by $\frac{1}{y_i}$

The weighted profile R factor, R_{wp} is

$$R_{wp} = \sqrt{\frac{\sum_i w_i (y_i - y_{ci})^2}{\sum_i w_i y_i^2}}$$

The profile R factor, R_p is

$$R_p = \sqrt{\frac{\sum_i |y_i - y_{ci}|}{\sum_i y_i}}$$

The statistically expected R value, R_{exp}, in which all deviations of the calculated pattern from the observed are due to statistical variations. R_{exp} is defined by:

$$R_{exp} = \sqrt{\frac{N_{obs} - N_{var}}{\sum_i w_i y_i^2}}$$

where N_{obs} and N_{var} are the number of observables and number of variables respectively.

A goodness of fit parameter, χ^2, is defined from the square of the ratio of R_{wp} and R_{exp}.
